# ADHD and Student Grades in Children from the ABCD Study^®^: A Twin and Siblings Study

**DOI:** 10.1007/s10519-026-10274-0

**Published:** 2026-07-24

**Authors:** Luis F. S. Castro-de-Araujo, Daniel Zhou, Mei-Hsin Su, Sydney Kramer, Robert Loughnan, Michael C. Neale

**Affiliations:** 1 Virginia Institute for Psychiatric and Behavioral Genetics, Virginia Commonwealth University, P.O. Box 980126, Richmond, VA 23298-0126 USA; 2 Department of Psychiatry, The University of Melbourne, Austin Health, Heidelberg, VIC Australia; 3 Population Neuroscience and Genetics, University of California, San Diego, La Jolla, USA

**Keywords:** ADHD, Educational attainment, MR-DoC

## Abstract

Attention-deficit/hyperactivity disorder (ADHD) symptoms are strongly associated with lower academic performance, yet the nature of this association remains uncertain. We analyzed data from twins and siblings in the Adolescent Brain Cognitive Development (ABCD) Study^®^ to examine the relationship between ADHD symptoms and school grades using genetically informed models. ADHD symptoms were assessed via the Child Behavior Checklist, and grades were caregiver-reported for the previous school year. Both phenotypes were modeled as ordinal variables. We applied Direction of Causation (DoC) and Mendelian randomization extensions (MR-DoC and MR-DoC2) incorporating polygenic risk scores for ADHD and educational attainment. DoC and MR-DoC comparisons did not distinguish directionality (e.g., DoC forward path estimate = − 0.16, 95% CI [− 0.26, − 0.07]; reverse path = − 0.30, 95% CI [− 0.63, − 0.17]). In MR-DoC2, estimates differed in precision: ADHD-to-grades path = − 0.31, 95% CI [− 0.62, − 0.11]; grades-to-ADHD path = − 0.60, 95% CI [− 1.20, 0.03]. Sex-limitation tests indicated no moderation by sex.

**Supplementary Information:**

The online version contains supplementary material available at 10.1007/s10519-026-10274-0.

## Introduction

Attention-Deficit/Hyperactivity Disorder (ADHD) is one of the most prevalent externalizing neurodevelopmental disorders, affecting from 7.2% (95%CI 6.7–7.8) (Thomas et al. 2015) to 7.6% (95%CI 6.1–9.4%) of all children and 5.6% (95%CI 4.8–7%) of adolescents (Salari et al. 2023). Inattention, hyperactivity and impulsivity are the main symptoms of ADHD, which is frequently comorbid with learning disorders, autism, conduct disorder, oppositional defiant disorder, and obsessive-compulsive disorder (Fayyad et al. 2017; Sousa et al. 2020). ADHD is also genetically correlated with autism spectrum disorder and depression (Demontis et al. 2019; Loughnan et al. 2022). Additionally, significant twin and SNP-based genetic correlations have been reported between ADHD and disruptive childhood behaviors, including conduct disorder and oppositional defiant disorder (Demontis et al. 2023; Tesli et al. 2024).

ADHD symptoms are highly heritable, apparently due to multiple genetic loci, each with a small effect (Faraone and Larsson 2019). Twin studies found a 9-fold increased risk of ADHD in siblings of probands compared to siblings of controls, corresponding to a sibling correlation of 0.3 (Chen et al. 2008; Faraone and Larsson 2019). Twin studies also found a heritability of 80% (Chen et al. 2017), and both twin and adoption studies suggest minimal shared environmental effects (Sprich et al. 2000). A male-to-female prevalence ratio has been reported as 2:1 (Sousa et al. 2020), however heritability differences across sexes were not found (Eaves et al. 1997; Martin et al. 2018; Vink et al. 2012). This discrepancy may reflect differential teacher reporting of ADHD symptoms by sex (Derks et al. 2007). This difference suggests that males are more likely to be identified with ADHD symptoms than females by external observers. In turn this may be due to differences in clinical characteristics, with males showing more hyperactivity and impulsivity than females (Biederman et al. 1999). Additionally, there is evidence of a sex-of-proband effect, with male relatives of female probands having higher genetic risk than female relatives of male probands (Rhee et al. 1999; Rhee and Waldman 2004; Taylor et al. 2016). There is no evidence of differential heritability of ADHD between males and females (Larsson et al. 2014), and the genetic correlation for ADHD between sexes is close to one (Taylor et al. 2016). Altogether, these findings suggest that the genetic factors influencing ADHD are the same in males and females, but females express the symptoms in a less disruptive fashion. It is currently unclear whether these differences result in differential effects on the association between ADHD and other phenotypes, or more specifically on educational performance.

ADHD symptoms are consistently associated with lower academic achievement (Boomsma et al. 2010; Demontis et al. 2019). However, these correlational studies are unable to establish a causal relationship between EA and ADHD. For causal inference, one usually needs a randomized intervention design, typically a randomized controlled trial (RCT). RCTs in children are rarely feasible due to ethical and logistical constraints. Mendelian randomization (MR) studies offer an alternative method to assess causation when RCTs are not feasible. MR analyses require a genetic variant that correlates enough with the exposure to avoid weak instrument bias (Bowden et al. 2015). In its simplest form, the comparison of GWAS with outcome and GWAS with exposure, MR also requires the assumption that the variant correlates with the outcome exclusively via the exposure variable, which is also known as the no horizontal pleiotropy or exclusion-restriction assumption. If this assumption holds, the genetic variant may be considered to be an instrumental variable. Besides MR, other alternatives to RCTs are causal inferential extensions of the classical twin design and cross-lagged panel models. For example, the Direction of Causation (DoC) models (Heath et al. 1993) use cross-twin cross-trait correlations to infer whether one phenotype causally influences another. The differences in genetic and environmental covariance between traits are used to test competing directional hypotheses. This model has been extended (i) with multiple indicators (Araujo et al. 2025; Gillespie et al. 2003), (ii) with polygenic scores as instrumental variables (MR-DoC) (Minică et al. 2018), improving robustness to horizontal pleiotropy, and (iii) with bidirectional causal inference with full background confounding through additive genetic (A), common environment (C) and unique environmental (E) covariances (Castro-de-Araujo et al. 2023b; 2025b).

Demange et al. (2023) examined the relationship between educational attainment (EA) and psychiatric disorders using two complementary approaches: within-sibship analyses in adults of Dutch national registry data and two-sample Mendelian randomization (MR). The within-sibship design controlled for shared familial factors, while MR leveraged genetic instruments to test causality in both directions. Within-sibship analyses supported a protective effect of EA on ADHD, with odds ratios per year of education in the range of 0.81–0.98. MR analyses suggested a bidirectional relationship: higher genetic liability to ADHD was associated with reduced EA (IVW ß= −0.38, *P* < 0.004), and conversely, higher EA liability lowered ADHD risk (IVW OR = 0.73, *P* < 0.004). These results are consistent with prior two-sample MR studies reporting reciprocal effects between EA and ADHD (Dardani et al. 2021; Michaëlsson et al. 2022).

MR studies have become very popular in situations where RCTs have limited use (Lewis and Vassos 2020). MR has four key assumptions: (1) exchangeability, which means that the variant is not associated with a confounding factor in the relationship between the exposure and outcome; (2) exclusion restriction, which states that the variant only affects the outcome through the exposure; (3) relevance, indicating that the instruments are sufficiently predictive of the exposure; and (4) there is no reverse causation from outcome to exposure. Evaluating whether the last three assumptions hold can often be challenging. First, the exclusion restriction assumption (also known as no horizontal pleiotropy) states that the genetic variants affect the outcome exclusively via the exposure variable. When the underlying biological mechanisms are well understood, such an assumption may be reasonable. For complex traits such as ADHD, there is a general lack of understanding of the pathways from SNP variant to outcome phenotype. It is therefore important to validate this assumption. Second, for complex traits such as ADHD the SNP variants affecting the outcome trait, are expected to be very weak. Most mental disorders are highly polygenic with each variant exerting a tiny effect on an outcome phenotype (Abdellaoui and Verweij 2021). Thus, there is a high risk of encountering biases due to weak instruments while applying MR to complex traits. While this problem may seem to be circumvented by the use of a weighted sum of the variant’s effects, such as a polygenic risk score (PRS), the opportunities for violation of the exclusion restriction assumption rapidly multiply.

The no horizontal pleiotropy assumption is thought to be implausible with respect to mental disorder phenotypes. Pleiotropy can be classified as vertical or horizontal. Vertical pleiotropy occurs when a variant has a causal effect on a risk factor or exposure, which then causes the outcome. It is explicitly modelled in MR and does not bias estimation. Horizontal pleiotropy occurs when a genetic variant has a causal effect on the outcome that is not mediated by the exposure variable. While such effects are often called ‘direct’ (partly because they are modelled with a single regression parameter of outcome on SNP variant or PRS), these alternative pathways from genetic variant to outcome phenotype would generally involve other mediating variables. To complicate things further, SNP variants are typically in linkage disequilibrium (LD) with other variants, which may have causal effects on the outcome. This type of pleiotropy is pervasive and considered part of the genetic architecture of complex traits (Jordan et al. 2019). Because SNPs are often in linkage disequilibrium (LD) with other variants that may influence the outcome, LD poses a major challenge for Mendelian randomization and can bias causal estimates (Verbanck et al. 2018).

To address these limitations, we leverage ABCD Study^®^ twin and sibling data using three complementary approaches that differ in their assumptions about pleiotropy and confounding. First, the classical Direction of Causation (DoC) model uses cross-twin cross-trait correlations to test whether ADHD symptoms or educational performance have causal effects on each other (Heath et al. 1993). Second, MR-DoC integrates polygenic scores as instrumental variables into the twin design, improving robustness to horizontal pleiotropy (Minică et al. 2018). Finally, MR-DoC2 extends this framework to allow bidirectional causal inference while accounting for genetic and environmental covariances (Castro-de-Araujo et al. 2023b,2025b).

Previous twin-based evidence supports the plausibility of a direct effect of ADHD on academic outcomes. For example, De Zeeuw et al. (2017) reported a significant direct path from ADHD symptoms to educational achievement in Dutch twins (β = −0.42, χ²(1) = 4.47, *p* = 0.034), even after controlling for genetic and environmental correlations. However, their design could not fully resolve bidirectionality, thus the need for models that explicitly test both directions.

The present study aims to clarify whether ADHD symptoms influence educational performance in childhood, or vice versa, when horizontal pleiotropy and reverse causation are considered. Using instrumental-variable extensions of twin designs applied to twin, independent-sibling, and singleton data from the ABCD Study^®^ (Haist and Jernigan. 2023), we test both directions of effect. Based on prior studies using Mendelian randomization, we anticipate the presence of bidirectional associations, though we expect no sex moderation of the effect sizes.

## Methods

### Data Source

Data were obtained from the release v5.0 of the Adolescent Brain Cognitive Development (ABCD) Study (Haist and Jernigan 2023), a large-scale, longitudinal study that aims to track the development of over 10,000 children across the United States. The study collects data on various domains, including neuroimaging, genetics, cognitive function, psychopathology, substance use and environmental factors (Maes et al. 2022). We used the sample of twins and siblings in the ABCD Study, more specifically only those for whom data were available from the follow-up year 3 of the study totalling 1,523 complete pairs (Table 1, header values). Incomplete pairs or singletons added another 183 rows in the analyses (DoC and sex limitation tests), amounting to 1,706 rows. Zygosity assignment was based on the calculated genetic correlation between the twins (Conomos et al. 2016). Sibling and singleton data were also used in the analyses, but no trios or siblings of twins were included due to their scarcity. The rationale for using the follow-up year 3 was to strike a balance between the age at which symptoms are present while avoiding the attrition that happened in later waves due to the pandemic. ABCD’s follow-up year 4 was particularly affected by missed visits. PRSs were calculated based on summary statistics from a GWAS that included Europeans only and the MR-DoC and MR-DoC2 analyses were restricted to individuals empirically assigned as European ancestry (*n* = 1,071) (Peterson et al. 2017). The DoC models were applied to the whole sample including all ancestral groups. In the ABCD Study, opposite-sex pairs were not ascertained, that is, they were not part of the study design but haphazardly recruited. The MZ, DZ, and sib pair polychoric correlations are reported in Table 1, stratified by sex, together with the means and standard deviations (SDs) for the key variables and standard errors (SE) for the correlations. Point estimates and maximum likelihood confidence intervals are reported for all models (Table 5).

**Table 1:** Sample description

Variable/Group correlations (complete pairs)	Mean	SD	NA%	rMZFF (169)	rMZMM (173)	rDZFF (244)	rDZMM (229)	rDZOS (57)	rSIBFF (161)	rSIBMM (163)	rSIBOS (327)
Grades (1482/224)	M: 0.009/F: 0.129		7.62	0.85 (0.03)	0.76 (0.04)	0.45 (0.06)	0.59 (0.05)	0.7 (0.08)	0.18 (0.08)	0.54 (0.07)	0.29 (0.06)
CBCL ADHD (1132/574)	M:0.083/F: −0.05		25.47	0.58 (0.07)	0.63 (0.06)	0.2 (0.08)	0.25 (0.08)	−0.06 (0.2)	0.42 (0.1)	0.36 (0.1)	0.27 (0.08)
C1 (1200/506)	0	0.03	24.97	0.93 (0.01)	0.9 (0.02)	0.97 (0)	0.74 (0.03)	0.62 (0.09)	0.93 (0.01)	0.89 (0.02)	0.81 (0.02)
C2 (1200/506)	0	0.01	24.97	0.92 (0.01)	0.96 (0.01)	0.71 (0.04)	0.56 (0.05)	0.78 (0.06)	0.53 (0.07)	0.61 (0.06)	0.54 (0.05)
C3 (1200/506)	0	0.01	24.97	0.49 (0.07)	0.54 (0.06)	0.68 (0.04)	−0.05 (0.07)	0.49 (0.11)	0.17 (0.09)	0.23 (0.08)	0.36 (0.06)
EUR EA PRS (945/761)	0	1	40.91	0.99 (0)	1 (0)	0.45 (0.07)	0.56 (0.06)	0.52 (0.12)	0.33 (0.09)	0.43 (0.08)	0.56 (0.05)
EUR ADHD PRS (945/761)	0	1	40.91	0.95 (0.01)	0.99 (0)	0.46 (0.06)	0.5 (0.06)	0.7 (0.09)	0.6 (0.07)	0.6 (0.07)	0.52 (0.05)

ABCD Study^®^ twin pairs at follow-up year 3 means (estimated from the latent distribution), standard deviations (SD), and group polychoric correlations with standard errors in parentheses. Header counts in parentheses are pairs per group; row counts in parentheses are (total pairs/incomplete pairs) for that variable across the full dataset. NA%, missingness

### Instruments and Polygenic Score Calculation

The Child Behavior Checklist (CBCL) ADHD subscale was used to evaluate ADHD symptoms and their progression over time. This subscale, which is completed by the participants’ caregivers, includes questions regarding inattention, hyperactivity, and impulsivity (Achenbach 2001). Widely employed for assessing child and adolescent behavior problems, the CBCL ADHD subscale assesses items corresponding to ADHD symptoms and produces dimensional scores aligned with DSM symptom domains (Achenbach 2013). In comparison to the reference standard of clinical evaluation by a qualified professional, the CBCL has been reported to have a sensitivity of 0.77 and specificity of 0.73 for diagnosing ADHD (Chang et al. 2016). The DSM-oriented CBCL scales generally exhibit a high Cronbach’s alpha of 0.92, while the CBCL ADHD subscale has been reported to have an area under curve of 0.75 for diagnostic efficiency (Jiang et al. 2023). This variable was recoded as a 8-category ordinal for model-fitting. Collapsing some categorical levels was required because a few levels had very few observations, which gave poor threshold resolution. The adjacent-level pooling algorithm by level frequency is available publicly in the umx R package Castro-de-Araujo et al. (2026).

The second phenotype of interest was the youths’ school grades in the previous year, as reported by their caregiver. This variable was coded so that 1 corresponded to A + and 12 corresponded to an F. This variable was reversed, so larger values represented better performance, and included in the models recoded as an 7-category ordinal using umx (Table S1).

PRSs were calculated from the latest GWAS for each phenotype. ADHD liability PRSs were estimated with summary statistics from Demontis et al. (2023). PRSs for educational attainment were calculated using summary statistics from Okbay et al. (2022) . Quality Control (QC) on the GWAS summary statistics was performed following standard guidelines (Choi et al. 2020), which included filtering out: SNPs with minor allele frequencies (MAF) < 0.01, INFO scores < 0.8, duplicate SNPs, and those that were ambiguous (ex A-T or G-C variants). QC of genotypic data in the target ABCD sample was also performed according to standard guidelines and included filtering out SNPs with a MAF < 0.01, Hardy-Weinberg-Equilibrium Fisher’s Exact Test p-values < 10^− 6^, and missingness rates > 0.01. Individual samples with genotype missingness rates > 0.01 were also excluded. We then used PRS-cs (Ge et al. 2019) to infer posterior SNP effect sizes which were then used to compute individual level PRS in the target ABCD study samples using PLINK 1 (Purcell et al. 2007). To account for LD, an external European LD reference panel constructed from the UK Biobank data was used as an input for PRS-cs. For PRS optimization, PRS-cs estimates a global shrinkage parameter phi, which by default is automatically learned from the data using a fully Bayesian approach, to produce posterior SNP effect size estimates with the best predictive performance. PRSs were computed in ABCD samples of European ancestry, as GWAS summary statistics from non-European ancestry groups were not available.

### Covariates

Sex was included as a covariate. In order to control for population stratification, the first six principal components (PCs) were included as covariates. These were within-ancestry PCs calculated in-house for the European sample from the ABCD study^®^ using PC-AiR (Conomos et al. 2015). Only six were included because the last PCs were not correlated to either phenotype.

### Data Analysis

We used R version 4.2.2 (https://cran.r-project.org/) in all further analyses, including the OpenMx and umx packages used for SEM (structural equation modeling) analyses (Castro-de-Araujo et al. 2026; Neale et al. 2016). Parameter estimation used full information maximum likelihood (FIML) with the Sequential Least Squares Quadratic Programming (SLSQP) optimizer (Kraft 1988), a constrained nonlinear solver suitable for SEM models with bounds and equality constraints. We verified that the models were identified using the OpenMx utility mxCheckIdentification (Hunter et al. 2021). The high computational costs of analyzing both phenotypes as ordinal categorical variables required the use of 23 CPUs from the VIPBG cluster (vipbg.vcu.edu/resources/computational-facilities). All tests considered an alpha of 0.05, although we focus more on effect size than significance, due to the large sample size of ABCD (Dick et al. 2021).

### Model Specification

**Fig. 1:**
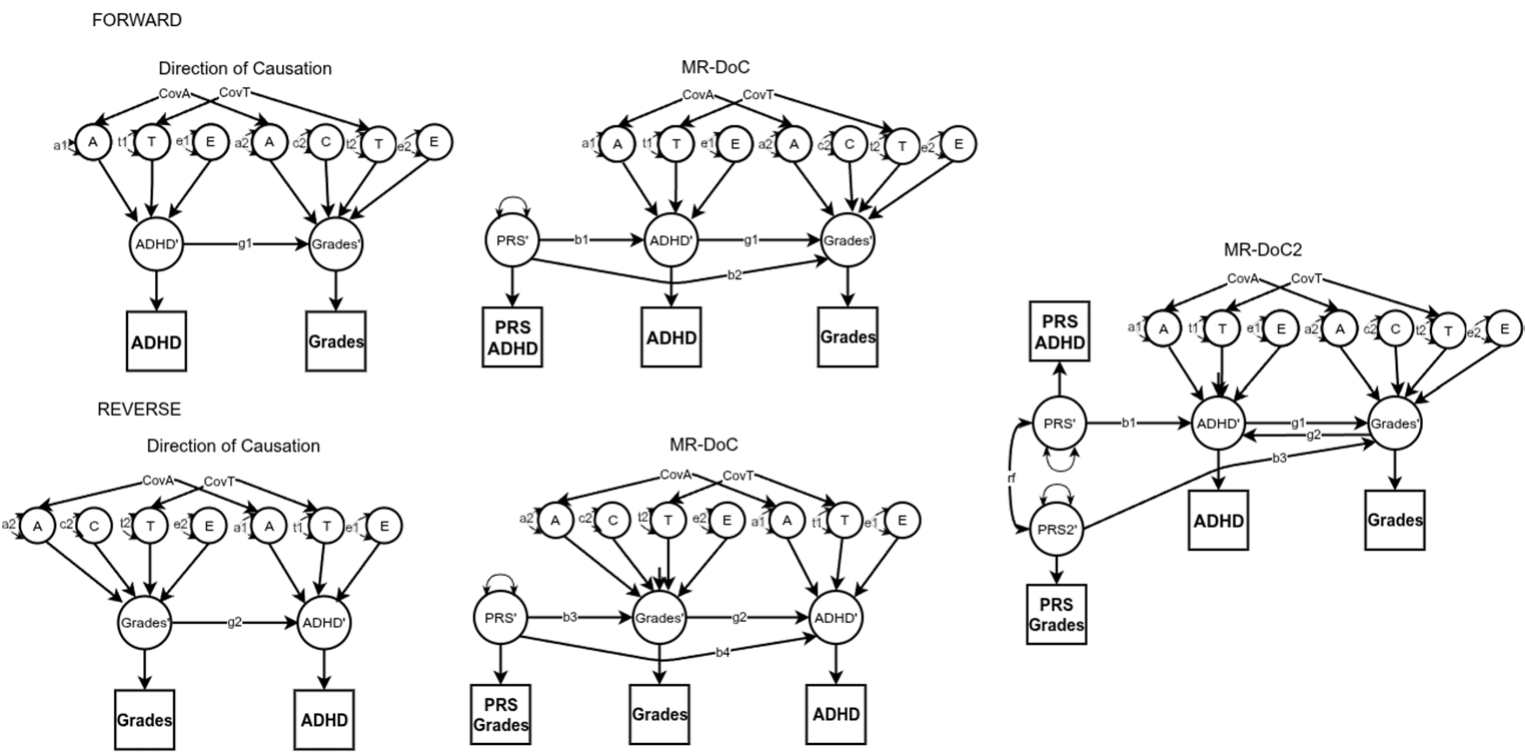
DoC, MR-DoC, and MR-DoC2 model specifications for a single member of a twin pair. The genetic cross-twin correlations are 1 for MZs and 0.5 for DZs and the shared environmental variance cross-twin correlations are 1 for both MZs and DZs (not shown). The models include the effects of additive genetic (A), common environment (C), being born together (T), and specific environment (E) factors for both traits, and their effects may correlate (parameters *covA*, covC, *covT*, and covE). The latent variables Grades’ and ADHD’ are not required for identification, nor are the scaling variables PRS1’ and PRS2’, but they are retained to be consistent with Castro-de-Araujo et al. (2025b, 2023b) papers

Three statistical models are used. The Direction of Causation (DoC) model (Fig. 1), a classic model that uses cross-twin cross-trait correlations to assess direction of causation between two phenotypes (Heath et al. 1993), and its extensions: MR-DoC, which adds a Mendelian randomization component (Minică et al. 2018); and MR-DoC2, which extends MR-DoC by allowing bidirectional causal inference in the presence of background confounding (Castro-de-Araujo et al. 2026, 2025b). Figure 1 shows path diagrams of all three models. MR-DoC allows for causal estimation without bias from horizontal pleiotropy, when one genetic locus directly affects both exposure and outcome, a common limitation from classic MR. MR-DoC2 accounts for indirect horizontal pleiotropy, when one genetic locus affects both exposure and outcome via linkage disequilibrium.

The DoC model is designed for data collected from relatives, and when these are MZ and DZ twin pairs it allows typical ACE decomposition. The phenotypic variances are decomposed into components associated with additive genetic (A), shared environmental (C) and unique environmental (E) variances. This research design cannot jointly estimate dominance genetic variance (D), which is confounded with (C). Adoption and twin studies have found that there is little shared environmental variance in ADHD (Sprich et al. 2000), so it is often modeled as an AE phenotype. ACE reduction tests confirmed what is found in the literature, and the ADHD symptoms phenotype is better modelled with an AE decomposition, whereas the school grades were better modelled as ACE (Table 2). For identification reasons the DoC model can only estimate three of the five paths (*g1*,* g2*,* covA*,* covC*, and *covE*); the specification used here drops *g2* (reciprocal causal path) and *covE* (specific environmental confounding or correlated errors of measurement; Fig. 1). Therefore, the DoC model will be estimated a second time in order to assess the reverse direction, with no reciprocal direction being tested.

**Table 2:** ACE reduction

Model	a	c	e	d	EP	Δ Fit	Δ df	*p*	AIC	Δ AIC
CBCL ADHD										
ACE	0.59	0	0.41		9				4956	0
ADE	0.31		0.38	0.29	9	−1.67	0		4954	−1.67
**AE**	**0.59**		**0.41**		**8**	**0**	**1**		**4954**	**−2**
Grades past year										
**ACE**	**0.42**	**0.43**	**0.15**		**6**				**3159**	**0**
ADE	0.86		0.14	0	6	23.87	0		3183	23.87
AE	0.86		0.14		5	23.87	1	< 0.001	3181	21.87

Standardized variance components. CBCL ADHD most parsimonious model included only A and E variance components. CBCL, Child Behavior Checklist. Best fitting models in bold

DoC models have been extended with PRS to allow for Mendelian randomization with various combinations of horizontal pleiotropy and background confounding. Minica et al. (2018) proposed MR-DoC (Fig. 1), which is an extended direction-of-causation model for twin data that uses polygenic scores as instrumental variables in MR. This model improves causal inference, which is robust to horizontal pleiotropy in some specifications. The *b2* path (Fig. 1) models the direct effect of the PRS on the outcome, therefore controlling for horizontal pleiotropy, whereas the path g1 is the causal path. The model is identified with five of the six paths (*b1*,* b2*,* g1*,* covA*,* covC*, and *covE*); the specification used here retains b2, as we are interested in controlling for horizontal pleiotropy, but dropped *covE*. Dropping *covE* when its true value is not zero biases the estimates of the causal path (g1) in the cases where *covE* does not equal zero. However, this bias is small (Castro-de-Araujo et al. 2025b, 2023b). CovC is not specified, as ADHD was modeled as AE.

The MR-DoC2 model is an extension to MR-DoC in which both traits have a PRS, thus allowing estimation of bidirectional causal paths with cross-sectional data. Each PRS works as an instrumental variable for each of the phenotypes in the model (Fig. 1). Pleiotropy is partially addressed by the model, but instead of the more direct specification of the b2 path in MR-DoC, it is specified indirectly as the paths b1*rf and b3*rf. Additionally, MR-DoC2 is identified with full ACE background confounding control (covA, covC, and covE; Fig. 1). The causal paths are g1 and g2. For all models, a T variance component (and a covT covariance) was added to capture variance that is due to being born together, because sibling pairs were added to this model and their correlations differ from those of DZ pairs (Table 1).

Finally, ordinal variables were specified using the Mehta-Neale approach of fixing the first two thresholds (Mehta et al. 2004) to obtain identification (Table S1) and all covariates were included in the model as definition variables (Neale et al. 2016).

## Results

Bivariate ACE reduction was performed using likelihood ratio tests to compare progressively more parsimonious models. ADHD symptoms were best fit by an AE model with variance components A = 0.59 and E = 0.41 (AIC = 4,954), whereas school grades were best modeled by an ACE model with A = 0.42, C = 0.43, and E = 0.15 (AIC = 3,159). All subsequent analyses were adjusted to these decomposition patterns (Table 2).

Because siblings were included and their correlations were higher than DZ correlations for ADHD symptoms but lower than DZ correlations for grades (Table 1), the models incorporated a T variance component to capture variance attributable to being born together (Fig. 1). For school grades, the MZ twin correlations were 0.85 for female–female pairs and 0.76 for male–male pairs, while for ADHD symptoms, the MZ correlations were 0.58 for females and 0.63 for males (Table 1). These patterns are consistent with prior literature (De Zeeuw et al. 2017).

Next, we formally tested sex limitation. Table 3 presents variance components for each variable by sex. There were substantial mean differences between sexes as seen in the estimated latent means in Table 1, but no meaningful differences in variance between males and females (Table 3). To further evaluate scalar sex moderation, we compared nested models where cross-sex correlations for A and C were constrained to equality. Likelihood ratio tests showed no significant decrement in fit when rA and rC were fixed to zero, indicating no evidence of sex limitation (Table 4).

Next, the target models were fitted to the data. Parameter estimates are unstandardized. The DoC model did not provide enough information to resolve directionality. The ADHD to Education causal path (g1) was estimated at − 0.16 [95% CI: −0.26, − 0.07], and the reverse path (g2) at − 0.30 [− 0.63, − 0.17] (Table 5). A likelihood ratio test comparing forward and reverse DoC models showed no appreciable difference (both have 27 estimated parameters [EP], ΔFit = 0.242, AIC DoC reverse = 12,330, ΔAIC = 0.242), indicating insufficient information to determine direction.

**Table 3:** Sex-specific variance-component estimates

	am	af	tm	tf	cm	cf.	em	ef
CBCL ADHD	0.59	0.584	0.002	0.02	0	0	0.362	0.405
Grades	0.61	0.661	0.048	0.17	0.15	0.002	0.182	0.166

Variance components by sex (m, males; f, females), variables treated as ordinal

**Table 4:** Sex-limitation model comparisons

Model	EP	Δ Fit	Δ df	*p*	AIC	Δ AIC	Compare with Model	Fit units
Scalar	31				17,922	0		−2lnL
Equated A	29	3.134	2	0.209	17,921	−0.866	Scalar	−2lnL
Equated A/C	28	7.502	3	0.058	17,924	1.502	Scalar	−2lnL
Equated A/C/T	26	9.864	5	0.079	17,922	−0.136	Scalar	−2lnL

Sex limitation test. The scalar model allows for free A/C/E across sex correlations. This model is then compared to nested models, with those correlations equated across sexes. No significant differences found. Variables treated as ordinal

**Table 5:** Unstandardized estimates for the DoC, MR-DoC, and MR-DoC2 models

Parameter	DoC	DoCr	MR-DoC	MR-DoCr	MR-DoC2
g1	− 0.16 [−0.26,−0.07]		−0.15 [−0.25,−0.06]		− 0.31[−0.62,−0.11]
g2		− 0.30 [−0.63,−0.17]		− 0.36 [−0.58,−0.15]	− 0.60 [−1.2,0.03]
a1	0.47 [0.27,0.71]	0.43 [0.25,0.66]	0.49 [0.30, 0.73]	0.47 [0.28,0.69]	0.41 [0.22,0.66]
t1	0 [−0.16,0.16]	0.04[−0.02,0.10]	− 0.06 [−0.22,0.09]	− 0.10[−0.25,0.03]	− 0.05 [−0.21,0.10]
e1	0.41 [0.31,0.56]	0.39[0.29,0.53]	0.39 [0.29,0.52]	0.37[0.27,0.49]	0.40 [0.29,0.57]
a2	0.27 [0.15,0.41]	0.29 [0.16,0.44]	0.26 [0.16,0.40]	0.26 [0.14,0.41]	0.22 [0.13,0.35]
c2	− 0.05 [−0.13,0.01]	− 0.05 [−0.13,0.01]	− 0.06 [−0.13,0.01]	− 0.04 [−0.12,0.02]	− 0.02 [−0.08,0.01]
t2	0.02 [−0.04,0.08]	0.04 [−0.02,0.11]	0.03 [−0.03,0.09]	0.05[−0.01,0.11]	0.01 [−0.05,0.07]
e2	0.14 [0.10,0.20]	0.15 [0.11,0.21]	0.14 [0.11,0.20]	0.16 [0.12,0.22]	0.15 [0.11,0.24]

Unstandardized parameter estimates and confidence intervals for causal paths and variance components for DoC, MR-DoC and MR-DoC2 models fitted to data on ADHD (1) and Educational performance (2) from the ABCD Study g1 is the causal path from ADHD to Education, and g2 is the reverse

For MR-DoC (EUR subsample), instrument strength was adequate (F-statistic = 37.3 for ADHD and 35.4 for EA/Grades). The ADHD to Education path estimate was − 0.15 [− 0.25, − 0.06], and the reverse path − 0.36 [− 0.58, − 0.15] (Table 5). Model comparison again showed no significant difference between directions (29 EP, ΔFit=−37.953, AIC MR-DoC reverse = 16,072, ΔAIC=−37.953, number of rows = 961).

Finally, MR-DoC2 was fitted to the European subsample. This step allows for directionality testing. The ADHD to Education effect was − 0.31 [− 0.62, − 0.11], while the reverse effect was − 0.60 [− 1.2, 0.03] (Table 5; Fig. 1). A likelihood ratio test confirmed that fixing g1 to zero significantly worsened model fit (ΔFit = 7.571, *p* = 0.006), supporting a significant causal effect of ADHD symptoms on educational performance but not vice versa (Table 6). Fixing g2 to zero did not worsen model fit, but fixing both causal parameters to zero also resulted in worse model fit (Table 6).

**Table 6:** Likelihood-ratio tests of unidirectional versus bidirectional causal effects (MR-DoC2)

Model	EP	Δ Fit	Δ df	*p*	AIC	Δ AIC	Compare with Model	Fit units
MRDoC2	44				20,716	0		−2lnL
No g1	43	7.571	1	0.006	20,721	3.724	MRDoC2	−2lnL
No g2	43	2.285	1	0.131	20,716	1.444	MRDoC2	−2lnL
No loop	42	10.203	2	0.006	20,722	5.331	MRDoC2	−2lnL

MR-DoC2 model fitted to the ABCD data on ADHD and Educational performance. Likelihood ratio tests (LRT) compared the full model against nested models fixing each of the causal paths (No g1, and No g2 models) or both paths (no loop model). Δ Fit, difference in −2lnL; df, degrees of freedom; p-value associated with the LRT; AIC, Akaike Information Criterion

The results support the hypothesis that there is a significant effect from ADHD symptoms to poor educational performance (g1), but not in the reverse direction (g2).

## Discussion

The analyses presented investigated the relationship between ADHD symptoms and grades using a twin design extended with instrumental variables, using data from children in the ABCD study. Our analyses indicate a significant causal effect of ADHD symptoms on educational performance in children, with an unstandardized estimate of − 0.31 (95% CI: −0.62, − 0.11) using MR-DoC2, a model robust to horizontal pleiotropy and shared familial confounding. Sex limitation was examined, which revealed no moderation by sex.

The findings provide further evidence for a relationship where ADHD symptoms adversely affect educational performance. This is consistent with previous findings from analysis using MR (Dardani et al. 2021; Demange et al. 2023; Michaëlsson et al. 2022). However, unlike those studies, which primarily relied on adult samples and standard MR assumptions, our approach explicitly accounts for horizontal pleiotropy and uses child-based data from twins and siblings. The reverse effect (educational performance → ADHD symptoms) was negative but non-significant, contrasting with some previous MR findings (Dardani et al. 2021; Michaëlsson et al. 2022). Although the ABCD study includes individuals of diverse ancestries, the PRSs were trained only in European GWAS, and therefore we were only able to use the data on individuals of European ancestry in the MR-DoC and MR-DoC2 analyses. This limitation highlights the need for multi-ancestry GWAS to improve generalizability.

Despite the differences of ADHD prevalence between sexes, no sex-specific genetic liability has been found in previous studies (De Zeeuw et al. 2017; Derks et al. 2007; Hinshaw 2018; Rhee et al. 1999; Rhee and Waldman 2004; Sousa et al. 2020). Consistent with these findings, sex-limitation tests revealed no significant differences in variance components between sexes in this sample. Furthermore, most prior MR studies focus on adult ADHD; our findings provide rare evidence for childhood ADHD effects on academic performance using genetically informed designs.

There are several potential limitations with this study that should considered. The model for twin data used here assumes additivity of genetic effects and absence of dominance, epistasis, assortative mating, and gene–environment correlation. Violations of these assumptions, particularly rGE, could bias estimates (Abdellaoui and Verweij 2021; Demange et al. 2021), for example Allegrini et al., (2022) has shown that rGE and interaction significantly affect the prediction of EA. Marital correlations for educational attainment are substantial, which may be due to social stratification or direct phenotypic assortment. Both stratification and assortment would inflate estimates of shared environment variance. Also, the classical twin design involves the equal environment assumption (EEA) that non-elicited environmental effects are equally correlated for MZ and DZ twin pairs. Although not tested here, this assumption has rarely been found to be violated (Kendler et al. 1993). The CBCL used in this study is not a diagnostic tool, although it is highly correlated with structured diagnostic instruments (Achenbach 2013). Year-3 follow-up was conducted during the COVID-19 pandemic, coinciding with ages at which ADHD prevalence typically peaks (Sousa et al. 2020). Because grades were caregiver-reported for the previous school year, the pandemic may also have affected the grades reported at this wave, potentially introducing additional error variance. The best fitting CBCL ADHD model specification included A, T, and E variances only. Although dropping variance components to identify the most parsimonious model is a field standard (Heath et al. 1993; Neale et al. 1994), in the context of instrumental-variables theory, it can reduce power to detect the causal estimates (Castro-de-Araujo et al. 2025a; 2025b; Maydeu-Olivares et al. 2019), because it fails to account for all background confounding . Although g1 is unstandardized, its magnitude suggests a meaningful impact of ADHD symptoms on grades.

Several environmental factors are associated with ADHD. Kim et al. (2020) report in an umbrella review that childhood eating disorder, low weight or preterm birth, low parental educational attainment, and exposure to lead (among others) are risk factors associated with ADHD. Parental educational attainment also predicts offspring educational attainment (Jami et al. 2021). Therefore, the educational environment that parents provide for their children may influence their children’s ADHD and education performance, which is a potential source of environmental and genetic confounding. Classic MR cannot account for this type of confounding, nor can MR-DoC (provided one intends to control for horizontal pleiotropy, retaining path *b2*), but it is controlled in MR-DoC2. This is a substantial advantage of our approach.

The present study contributes to evidence on the association between ADHD symptoms and educational performance using genetically informed models in a large, community-based sample. Unlike clinical cohorts, ABCD was not ascertained for ADHD, which reduces selection bias but also means symptom prevalence reflects the population. Our approach’s strength lies in modeling both phenotypes as ordinal variables under FIML and incorporating polygenic scores within twin designs, which improves robustness to horizontal pleiotropy and shared familial confounding. However, residual gene–environment correlation and unmeasured environmental factors (such as the COVID-19 pandemic) remain possible. Future work should include replication using unrelated individuals and longitudinal models with random intercepts to capture symptom persistence at the individual-level (Castro-de-Araujo et al. 2025a; Hawes et al. 2025; Singh et al. 2024), and explore gene–environment interactions (Boker et al. 2023) to better understand developmental dynamics.

## Supplementary Information

Below is the link to the electronic supplementary material.

Supplementary Material 1

## Data Availability

Data used in the preparation of this article were obtained from the Adolescent Brain Cognitive Development (ABCD) Study (https://abcdstudy.org), held in the NIMH Data Archive (NDA). This is a multisite, longitudinal study designed to recruit more than 10,000 children age 9-10 and follow them over 10 years into early adulthood. A listing of participating sites and a complete listing of the study investigators can be found at https://abcdstudy.org/consortium_members/.

## Code Availability

github.com/lf-araujo/2023-mrdoc2-EA-ADHD ^The scripts used for genetic ancestry assignment and genetic principal component analysis are available upon request^
