# Supplementary material for: ADHD and Student Grades in Children from the ABCD Study®: A Twin and Siblings Study
Source: Behav Genet. 2026 Jul 24;56(4):194–205. doi: 10.1007/s10519-026-10274-0 (PMC13421242; doi:10.1007/s10519-026-10274-0)
Supplement: Supplementary file 1 — Supplementary Material 1 [file 10519_2026_10274_MOESM1_ESM.docx]

# Supplement

# [Table](#table_pool)S1

| Variable | Pooled |
| --- | --- |
| Grades (n, %) |  |
| 1 — 8 (0.2) | 1 — 8 (0.2)  — *th* ***-1.102 (fixed)*** — |
| 2 — 29 (0.9) | 2 — 616 (19.2)  — *th* ***0.418 (fixed)***— |
| 3 — 33 (1.0) |  |
| 4 — 76 (2.4) |  |
| 5 — 105 (3.3) |  |
| 6 — 190 (5.9) |  |
| 7 — 183 (5.7) |  |
| 8 — 351 (10.9) | 8 — 351 (10.9)  — *th 0.252* — |
| 9 — 439 (13.7) | 9 — 439 (13.7)  — *th 0.272* — |
| 10 — 457 (14.2) | 10 — 457 (14.2)  — *th 0.421* — |
| 11 — 843 (26.3) | 11 — 843 (26.3)  — *th 0.491* — |
| 12 — 419 (13.1) | 12 — 419 (13.1) |
| NA — 77 (2.4) | NA — 77 (2.4) |
| CBCL ADHD (n, %) |  |
| 0 — 1076 (33.5) | 0 — 1076 (33.5)  — *th* **-0.195 (fixed)** — |
| 1 — 418 (13.0) | 1 — 418 (13.0)  — *th* **0.428 (fixed)** — |
| 2 — 278 (8.7) | 2 — 278 (8.7)  — *th* 0.309 — |
| 3 — 210 (6.5) | 3 — 210 (6.5)  — *th* 0.240 — |
| 4 — 152 (4.7) | 4 — 152 (4.7)  — *th* 0.240 — |
| 5 — 119 (3.7) | 5 — 119 (3.7)  — *th* 0.259 — |
| 6 — 95 (3.0) | 6 — 95 (3.0)  — *th* 0.249 — |
| 7 — 68 (2.1) | 7 — 177 (5.5) |
| 8 — 42 (1.3) |  |
| 9 — 21 (0.7) |  |
| 10 — 16 (0.5) |  |
| 11 — 14 (0.4) |  |
| 12 — 5 (0.2) |  |
| 13 — 10 (0.3) |  |
| 14 — 1 (0.0) |  |
| NA — 685 (21.3) | NA — 685 (21.3) |

Ordinal variable level pooling and empirical thresholds. For identification the first two thresholds are fixed.
